# When Pulmonary Embolism Opens the Door: Paradoxical Thalamic Stroke Through a Patent Foramen Ovale

**DOI:** 10.7759/cureus.110296

**Published:** 2026-06-05

**Authors:** Bader Jad Allah, Ibrahim D Al-Obaidi, Ahmed F Hasan, Abdulbaset E Elkkari

**Affiliations:** 1 Family Medicine, Seha Clinics, Abu Dhabi, ARE; 2 Academic Affairs, Tawam Hospital, Abu Dhabi, ARE; 3 Internal Medicine, Tawam Hospital, Abu Dhabi, ARE; 4 Pulmonary Medicine, Sheikh Tahnoon Medical City, Abu Dhabi, ARE

**Keywords:** deep vein thrombosis (dvt), paradoxical embolism, patent foramen ovale (pfo), pulmonary embolism (pe), thalamic infarct

## Abstract

Paradoxical embolism through a patent foramen ovale (PFO) is an uncommon but clinically important cause of acute ischemic stroke, particularly in the setting of pulmonary embolism (PE). We report a 46-year-old man who presented with bilateral grade C3-R (previously sub-massive) PE and subsequently developed focal neurological deficits, including word-finding difficulty. CT pulmonary angiography confirmed the diagnosis of PE, and transthoracic echocardiography with bubble study identified a PFO with right-to-left shunting. Contrast-enhanced brain MRI then demonstrated an acute left thalamic infarct consistent with paradoxical embolic stroke. Alternative etiologies were excluded. Management decisions regarding anticoagulation and PFO closure were made through a structured multidisciplinary process involving the patient. This case draws attention to the need for a high index of suspicion for paradoxical embolism when PE and PFO coexist with new neurological symptoms, and shows how early recognition, timely workup, and patient-centred decision-making together affect clinical outcomes.

## Introduction

Patent foramen ovale (PFO) is a persistent interatrial communication that fails to close after birth and is present in approximately 25% of adults [1,2,3]. Paradoxical embolism occurs when a venous thrombus passes from the venous to the arterial circulation through a right-to-left shunt, most commonly a PFO, bypassing pulmonary filtration and potentially causing stroke or other systemic arterial events [1,2]. We present the case of a 46-year-old man with extensive lower-extremity deep vein thrombosis (DVT) and bilateral grade C3-R pulmonary embolism (PE) who developed an acute ischemic stroke in the context of a documented PFO.

## Case presentation

A 46-year-old Egyptian man, employed as a driver, was brought to the emergency department by ambulance on 21/04/2025 after waking with a sudden onset of severe dyspnea.

His background included essential hypertension, mixed dyslipidaemia, heart failure with reduced ejection fraction (HFrEF; diagnosed at age 44 and previously treated with sacubitril/valsartan, which was discontinued at age 45 as claimed by the patient), and coronary artery disease with percutaneous coronary intervention to the left anterior descending artery at age 30, performed in Egypt. His medications on admission were dapagliflozin 10 mg daily, atorvastatin 40 mg daily, bisoprolol 2.5 mg daily, lisinopril 2.5 mg daily, and aspirin 75 mg daily. He had undergone right inguinal hernia repair in January 2025, and a left lower limb DVT diagnosed in March 2025 was managed conservatively with watchful waiting.

On arrival, he was tachycardic and markedly hypoxic, with oxygen saturation of 80% on room air. Asymmetric left lower-limb swelling was present on examination, raising immediate concern for recurrent DVT with PE. Bilevel positive airway pressure (BiPAP) was initiated for respiratory support.

Initial investigations showed markedly elevated high-sensitivity troponin and B-type Natriuretic Peptide (BNP) (Table 1), consistent with right ventricular strain and myocardial injury. Chest radiography showed a prominent right hilum. Venous Doppler ultrasound (Figure 1) demonstrated extensive subacute to chronic thrombosis of the left lower limb from the posterior tibial vein to the common femoral vein. CT pulmonary angiography (Figure 2) confirmed bilateral PE, with segmental and subsegmental filling defects in the right lower lobe pulmonary artery and subsegmental branches of the left lower lobe.

**Table 1:** Initial Laboratory Findings on Presentation to the Hospital *Reference ranges are per institutional laboratory standards. **Both laboratory values are reported in ng/L. The BNP reference range is less than 100 ng/L.

Laboratory Test	Result	Reference Range*	Interpretation
High-sensitivity Troponin	329.8 ng/L	<14 ng/L	Elevated
B-type Natriuretic Peptide (BNP)	663.0 ng/L	<100 ng/L**	Elevated

**Figure 1:**
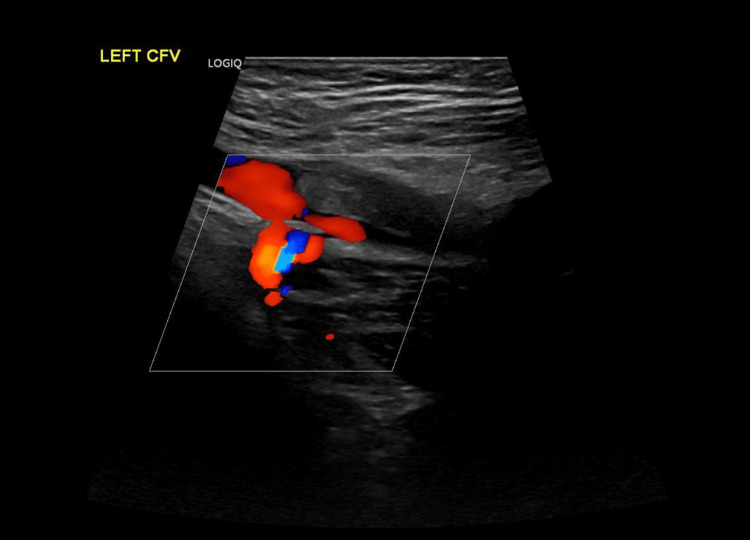
Color Doppler Ultrasound of the Left Common Femoral Vein (CFV) Demonstrating Deep Vein Thrombosis

**Figure 2:**
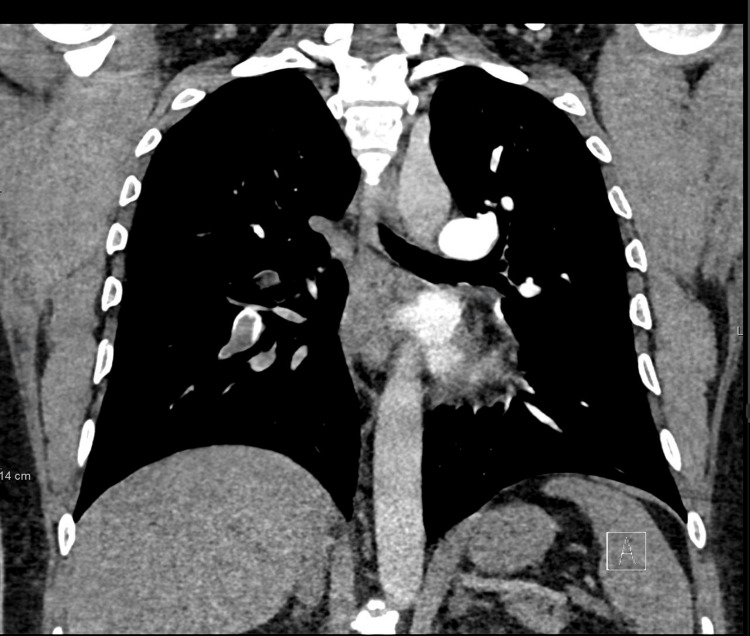
Contrast-Enhanced Computed Tomography of the Chest Demonstrating Bilateral Pulmonary Embolism

Transthoracic echocardiography (Figure 3) showed a dilated right ventricle with severely reduced systolic function. An agitated saline bubble study demonstrated early microbubble passage across the interatrial septum, confirming a PFO with right-to-left shunting. The absence of systemic hypotension in the context of right ventricular dysfunction established the diagnosis as bilateral grade C3-R PE. Therapeutic enoxaparin (1 mg/kg) was started, and the patient was admitted to the intensive care unit.

**Figure 3:**
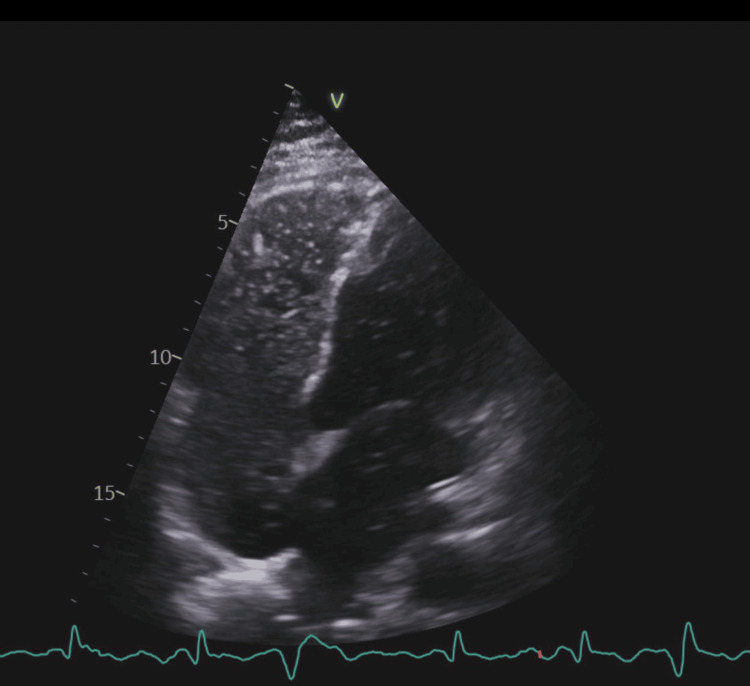
Transthoracic Echocardiography With Agitated Saline (Bubble Study) Demonstrating Right to Left Shunt Across a Patent Foramen Ovale

The following morning, he developed word-finding difficulty. Brain MRI (Figure 4) showed a focal diffusion-restricted lesion in the left thalamus consistent with acute ischemic stroke. A multidisciplinary team, including neurology, cardiology, and pulmonology, reviewed the case and concluded that the stroke was most likely caused by paradoxical embolism through the PFO.

**Figure 4:**
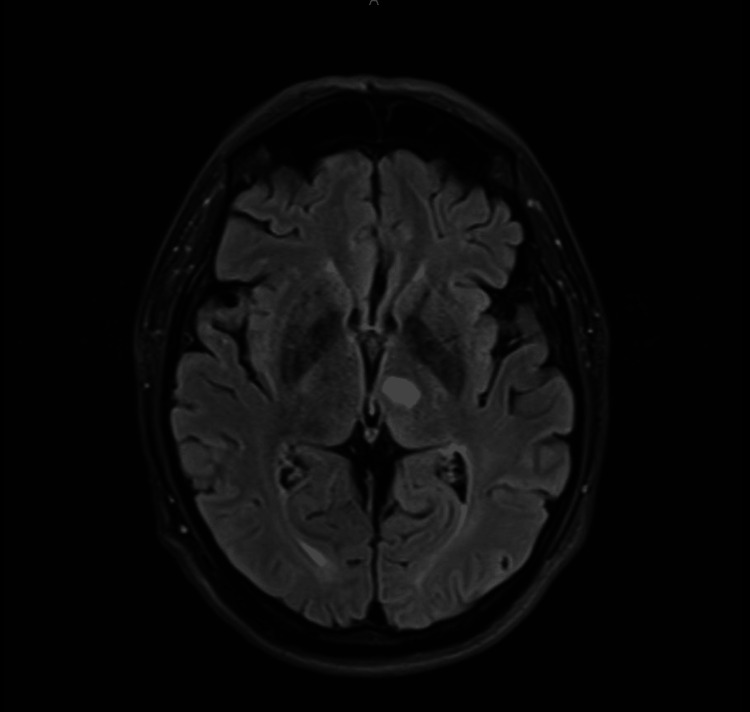
Brain Magnetic Resonance Imaging (Axial FLAIR Sequence) Demonstrating Acute Left Thalamic Infarction FLAIR: fluid-attenuated inversion recovery

The simultaneous presence of grade C3-R PE, acute thalamic stroke, and a PFO, each with its own therapeutic implications, made management genuinely complex. A formal meeting was convened with the patient and his next of kin, facilitated jointly by the attending physicians from neurology, cardiology, and pulmonology. The goal was to explain the diagnosis in plain terms, present the available options with their respective risks and benefits, and arrive at a treatment plan the patient could understand and accept.

Three decisions were discussed collaboratively. For inferior vena cava (IVC)filter placement, the patient was informed of both the intended benefit of reducing further embolic risk in the acute phase, given the extensive residual clot burden, and the long-term risks, before consenting to proceed. On anticoagulation, the team explained that therapeutic enoxaparin would continue acutely and transition to oral edoxaban at discharge, with aspirin discontinued in view of the independent indication for lifelong anticoagulation arising from recurrent unprovoked DVT and PE. The patient confirmed understanding and agreement. The question of PFO closure was the most involved of the three. The cardiology team explained the trial evidence directly: closure is beneficial in selected patients aged 60 or under with high-risk PFO anatomy, but when lifelong anticoagulation is already required for another reason, the additive benefit of closure is uncertain, as noted in current expert consensus [2,4,5]. The patient asked questions, involved his family, and agreed with the clinical team that urgent closure was not warranted and that outpatient elective evaluation would be the appropriate next step. The decision was reached jointly, not imposed.

Prior to discharge, the patient received detailed counseling on anticoagulation adherence and was transitioned to oral edoxaban. At follow-up, he was clinically stable, with full resolution of his neurological symptoms.

## Discussion

Paradoxical embolism was once thought extremely rare. Fewer than 128 cases had been reported before 1972, prior to the introduction of contrast echocardiography [6]. With better diagnostic tools, the picture changed. Among adults aged 18-60, roughly half of all cryptogenic ischemic strokes occur in the presence of a PFO, corresponding to an estimated 18,000 cases annually in the United States and 345,000 worldwide [4]. A pooled analysis of 23 case-control studies (1,154 stroke cases, 1,852 controls) found PFO significantly more common in cryptogenic stroke, with an odds ratio of 2.9 (95% CI 2.1-4.0) [7]. In patients under 55, this rises to 5.1 (95% CI 3.3-7.8), with a PFO-attributable fraction of roughly 80%, compared with 52% in older adults [7].

The core mechanism is straightforward: a venous thrombus crosses the interatrial septum when right-atrial pressure exceeds left-atrial pressure, which can occur during a Valsalva maneuver, coughing, or acute PE. In situ thrombus formation within the PFO tunnel, or on a coexistent atrial septal aneurysm, can also act as a source of arterial embolisation [8]. PE is a particularly potent trigger. When right-sided pressures rise acutely, even a small PFO can open and permit emboli to reach the systemic circulation. In a prospective cohort of patients with acute PE, approximately 40% had a PFO, and its presence independently predicted new ischemic brain lesions on MRI with an odds ratio of approximately 4.6, even in anticoagulated patients [9]. Case-level evidence supports the same conclusion: new neurological symptoms in the setting of DVT/PE, particularly in younger patients without atherosclerotic risk factors, should prompt urgent evaluation for paradoxical embolism [8,10]. Increased right-to-left shunting has also been documented in migraine with aura and obstructive sleep apnea, which points to transient shunt physiology as a mechanism beyond stroke [11,12].

Diagnosing paradoxical embolism requires satisfying three conditions: a venous embolic source, a right-to-left intracardiac shunt, and an arterial embolic event [2]. In this patient, all three were met. Meeting these three criteria, however, is not the end of the diagnostic process. Before a PFO can be held responsible, competing etiologies must be worked through systematically. American Heart Association/American Stroke Association (AHA/ASA) guidelines call for prolonged rhythm monitoring, vascular imaging for large artery atherosclerosis, hypercoagulability testing, and assessment for cardioembolic sources, including atrial fibrillation and endocarditis [13]. Continuous cardiac monitoring throughout the admission revealed no atrial fibrillation or other arrhythmia. Carotid and intracranial vascular imaging showed no significant atherosclerotic disease. Transthoracic echocardiography found no intracardiac thrombus, and neither the clinical picture nor laboratory findings pointed to infective endocarditis. The patient was young, without the atherosclerotic burden sufficient to explain a thalamic infarct, and had no prior history of paroxysmal palpitations. A thrombophilia screen was deferred to the outpatient setting, and a malignancy workup was considered in view of the recurrent unprovoked nature of his DVT. This methodical exclusion of competing mechanisms, combined with the temporal relationship between right ventricular strain and neurological symptom onset, makes paradoxical embolism the most defensible diagnosis. The diagnostic approach used here reflects current best practice: CT pulmonary angiography is the first-line test for confirming PE and assessing right-heart strain [1,2]; transthoracic echocardiography with agitated saline is a practical initial screen for PFO, though transesophageal echocardiography remains the reference standard for anatomical characterization [14,15]; transcranial Doppler provides a highly sensitive physiological assessment of shunt magnitude [15]; and brain MRI with diffusion-weighted imaging is the preferred modality for confirming acute infarction and identifying clinically silent embolic lesions [16].

An autopsy study of 965 structurally normal hearts found PFO in 27%, with prevalence falling from 34% in those under 30 to 20% in those over 80; mean defect size increased with age, consistent with preferential spontaneous closure of smaller defects [3]. This raises the central clinical question in any such case: is the PFO causally responsible, or incidental? The Risk of Paradoxical Embolism (RoPE) score addresses this directly. Patients scoring 9-10 have a PFO prevalence of 73-77% and an attributable fraction of 88%; those scoring below 3 have an attributable fraction of 0% [2]. The PASCAL (PFO-Associated Stroke Causal Likelihood)** **classification adds anatomical risk features, including large shunt size and atrial septal aneurysm, to categorize causality [2,4]. Randomized trial data support closure in patients aged 60 or under with high-risk anatomy; a meta-analysis of six major trials (3,740 participants) reported annual recurrent stroke rates of 0.47% after closure versus 1.09% with medical therapy alone (hazard ratio 0.41), and PASCAL "probable" patients had a 90% relative risk reduction. In patients with PASCAL "unlikely" classification, closure confers no benefit and adds procedural risk [2,4].

Current guidelines reflect this tiered approach. The European Stroke Organisation (ESO) (2024) recommends closure plus antiplatelet therapy for patients aged 18-60 with PFO-associated cryptogenic stroke and no identified alternative cause [17]. AHA/ASA (2021) considers closure reasonable (Class IIa) in patients aged 18-60 with non-lacunar stroke and high-risk PFO features [13]. The Society for Cardiovascular Angiography and Interventions (SCAI) (2022) guidelines address patients already requiring lifelong anticoagulation: closure can be considered, but the evidence is of very low certainty, and expert consensus acknowledges that the additive benefit of closure over full-dose anticoagulation remains unclear [5].

Several evidence gaps remain. No studies have quantified whether the severity of right ventricular strain or the degree of right-to-left shunting during grade C3-R PE predicts paradoxical stroke risk. The prognostic significance of PFO anatomy during acute PE has not been established. No randomized trials have addressed the management of PFO specifically in patients with grade C3-R PE [2,4,8]. These gaps matter in practice: they are what make protocol-driven management insufficient and shared decision-making necessary.

In this patient, the findings of extensive DVT, bilateral PE with right-heart strain, PFO with confirmed right-to-left shunting, and acute ischemic stroke together make a strong case for paradoxical embolism as the cause. Because he requires lifelong anticoagulation for recurrent DVT/PE, guidelines support anticoagulation as the primary strategy, with PFO closure left as an elective consideration subject to multidisciplinary review [2,4]. Pursuing outpatient evaluation for closure, as done here, is consistent with all major guideline frameworks. The structured shared decision-making process, in which the patient was fully informed of the evidence and its limitations, was both an ethical requirement and a practical component of management in a case with genuine uncertainty.

## Conclusions

Paradoxical embolism is an important and potentially underrecognized cause of acute ischemic stroke in patients with DVT and PE. New neurological deficits in the setting of acute venous thromboembolism should prompt evaluation for a PFO. Management calls for multidisciplinary input weighing anticoagulation requirements, recurrence risk, and the uncertain benefit of percutaneous closure in patients who already need lifelong anticoagulation. Structured consent and shared decision-making are not administrative steps: in cases where evidence is limited and the stakes are high, they are part of the clinical work itself. Early identification and treatment decisions made with the patient, not just for the patient, are what improve outcomes.
